# Comparison between arthroplasty and non-operative treatment for proximal humeral fractures: a systematic review and meta-analysis

**DOI:** 10.3389/fmed.2024.1436000

**Published:** 2024-09-06

**Authors:** Boyong Lai, Sheng Zhang, Junxi Pan, An Li, Ding Guo, Zhihua Peng, Qinghui Feng

**Affiliations:** The Affiliated Traditional Chinese Medicine Hospital, Guangzhou Medical University, Guangzhou, China

**Keywords:** arthroplasty, reverse shoulder arthroplasty, hemiarthroplasty, non-surgical treatment, functions, complications

## Abstract

**Background:**

The clinical efficacy of reverse shoulder arthroplasty (RSA), hemiarthroplasty (HA), and non-surgical management in the treatment of proximal humeral fractures (PHFs) is inconclusive. This systematic review and meta-analysis compared the clinical outcomes of arthroplasty and non-surgical management of PHFs.

**Methods:**

The databases of PubMed, Embase, Web of Science, and Cochrane Library were searched on 5 May 2023 for studies comparing arthroplasty and non-surgical treatment of PHFs. Both randomized controlled trials (RCTs) and non-randomized controlled trials (nRCTs), were included. Standard methodological quality assessments were conducted for both types of studies. The primary outcome was the Constant-Murley Score (CMS) after surgical or non-surgical treatment. Secondary study outcomes included the visual analog scale (VAS), range of motion, and complications. All functional scores and complications were subjected to subgroup and sensitivity analyses.

**Results:**

A total of four RCTs and six nRCTs were included in this study, which provided 508 patients in total for meta-analysis: 238 treated with arthroplasty and 270 treated non-surgically, of which 83 were treated with HA and 155 with RSA. All relevant information was collected, including functional scores, VAS, range of motion, and complications. The study found no significant difference in functional outcomes (mean difference, 2.82; 95% confidence interval, −0.49 to 6.14; *P* = 0.10; *I*^2^ = 77%) and complications (mean difference, 1.08; 95% confidence interval, 0.51–2.25; *P* = 0.85; *I*^2^ = 47%) between arthroplasty and non-surgical treatment. Both RCTs and nRCTs showed the same results. However, VAS scores were significantly lower in surgical treatment compared to non-surgical treatment. Subgroup and sensitivity analyses showed that RSA could achieve better functional scores than non-surgical treatment (mean difference, 6.00; 95% confidence interval, 1.97–10.03; *P* = 0.004; *I*^2^ = 0%), while the results for HA were not significant (*P* > 0.05).

**Conclusion:**

There were no significant differences in complications between arthroplasty and non-surgical treatment for PHFs. RSA could achieve better functional results than non-surgical treatment, while HA could only achieve better forward flexion.

## 1 Introduction

Proximal humeral fractures (PHFs) are the third most common fracture in older adults, accounting for 5–6% of all fractures. The incidence of PHFs increases with age and is higher in women (1). The mortality rate of PHFs is 1.68% within the 1st month, which is five times higher compared to the general population's mortality rate, and 7.83% within the 1st year, which is twice as high compared to the general population's rate.

Studies have shown that non-surgical treatment, along with factors such as increasing age, male sex, complex fractures, and low-energy trauma mechanisms, are risk factors for increased mortality. In contrast, arthroplasty is associated with the lowest risk of mortality (2). Walter's analysis of a registry of 47,979 patients with PHFs found that the 1-year mortality rate was significantly higher after non-operative treatment, at 16.4%, compared to a 7.4% mortality rate for those who underwent shoulder arthroplasty (3).

There are various treatment methods for PHFs, but the choice between surgical and conservative treatments remains controversial, especially for 3- or 4-part fractures. Current studies have shown that a surgical treatment does not result in better functional recovery and is equivalent to non-surgical treatment (4, 5). However, with advancements in technology, both HA and reverse shoulder arthroplasty (RSA) have been widely applied in treating PHFs. Despite the increasing incidence of PHFs over the past decade, non-surgical treatment continues to be a commonly used treatment option (6, 7). However, there is still no consensus on the use of arthroplasty for treating PHFs.

Therefore, this study aims to analyze the clinical outcomes and complication rates of arthroplasty, including HA and RSA, compared with conservative treatment for PHFs. We hypothesized that arthroplasty would yield similar outcomes to non-surgical treatment for PHFs.

## 2 Methods

To improve the reporting of systematic reviews and meta-analyses for RCTs and nRCTs, this report followed the guidelines published by the Preferred Reporting Items for Systematic Reviews and Meta-Analyses (PRISMA) and Meta-analysis of Observational Studies in Epidemiology (MOOSE).

### 2.1 Search strategy and eligibility criteria

On 5 May 2023, two reviewers (Lai. and Pan.) independently searched PubMed, EMBASE, Web of Science, and the Cochrane Library databases to identify all relevant studies. The search syntax is provided in Appendix S1. Both RCTs and nRCTs were included. After the removal of duplicates and screening of the titles and abstracts of the identified records, the studies were independently assessed based on their full texts. The eligibility criteria included studies comparing arthroplasty and non-surgical treatment with data on functional outcomes and complications. The exclusion criteria included letters, comments, case reports, non-English published publications, and the lack of full text. If a disagreement arose, a third expert (Feng.) intervened and made the final decision.

### 2.2 Data extraction

The reviewers independently extracted relevant data from the included studies. We collected the following data: the first author's name, journal, publication year, study design, study period, sample size, interventions, mean age, female ratio, duration of follow-up, fracture type, functional outcomes, and complications. Complications mainly included non-union, osteonecrosis, additional surgery, and other complications described in the original study.

### 2.3 Quality assessment

The two reviewers independently evaluated the methodological quality and risks of bias of the RCTs and nRCTs using the Methodological Index for Non-Randomized Studies (MINORS) (8). MINORS is a validated tool for assessing the methodological quality of observational studies and has been externally validated for RCTs through comparison with the CONSORT statement, making it suitable for meta-analyses involving differing study designs. The MINORS scores range from 0 to 24, with 0–8 points classified as low-quality literature, 9–16 points as medium-quality literature, and 17–24 points as high-quality literature. According to the MINORS scale, studies with a score of < 12 points were excluded from the meta-analysis. Disagreements were resolved by involving a third reviewer (Feng.).

### 2.4 Outcome measures

The primary outcome measure was physical function, assessed using the Constant-Murley Score (CMS). Secondary outcome measures included the visual analog scale (VAS) for pain, range of motion, and complications.

If available, other functional outcome measures, such as the Disabilities of the Arm, Shoulder, and Hand (DASH), the American Shoulder and Elbow Surgeons shoulder scores (ASES), and the EuroQol 5 Dimensions Questionnaire (ED-5Q), were also extracted. In addition, both HA and RSA were compared separately with non-surgical treatment. Both RCTs and nRCTs were analyzed independently.

### 2.5 Statistical analysis

All data were analyzed using Review Manager version 5.3. All continuous variables were converted to means and standard deviations when sufficient information was available. Dichotomous variables were presented as odds ratios (ORs) with a 95% CI. A *P*-value of < 0.05 was considered statistically significant. When the data exhibited heterogeneity (*P* < 0.1 or *I*^2^ > 50%), a random-effects model was used for the meta-analysis. Otherwise, a fixed-effects model was used. Publication bias was evaluated using a funnel plot.

All analyses were stratified by study design, with RCTs and nRCTs analyzed separately and included in both designs. RSA and HA were also analyzed separately.

### 2.6 Subgroup analyses and sensitivity analyses

Subgroup analyses were conducted for RCTs, nRCTs, RSA, and HA, including CMS, VAS, forward flexion, external rotation, and complications. To address study heterogeneity, sensitivity analyses were performed on studies with high consistency using the fixed-effects model.

## 3 Results

### 3.1 Literature search

**Figure 1:**
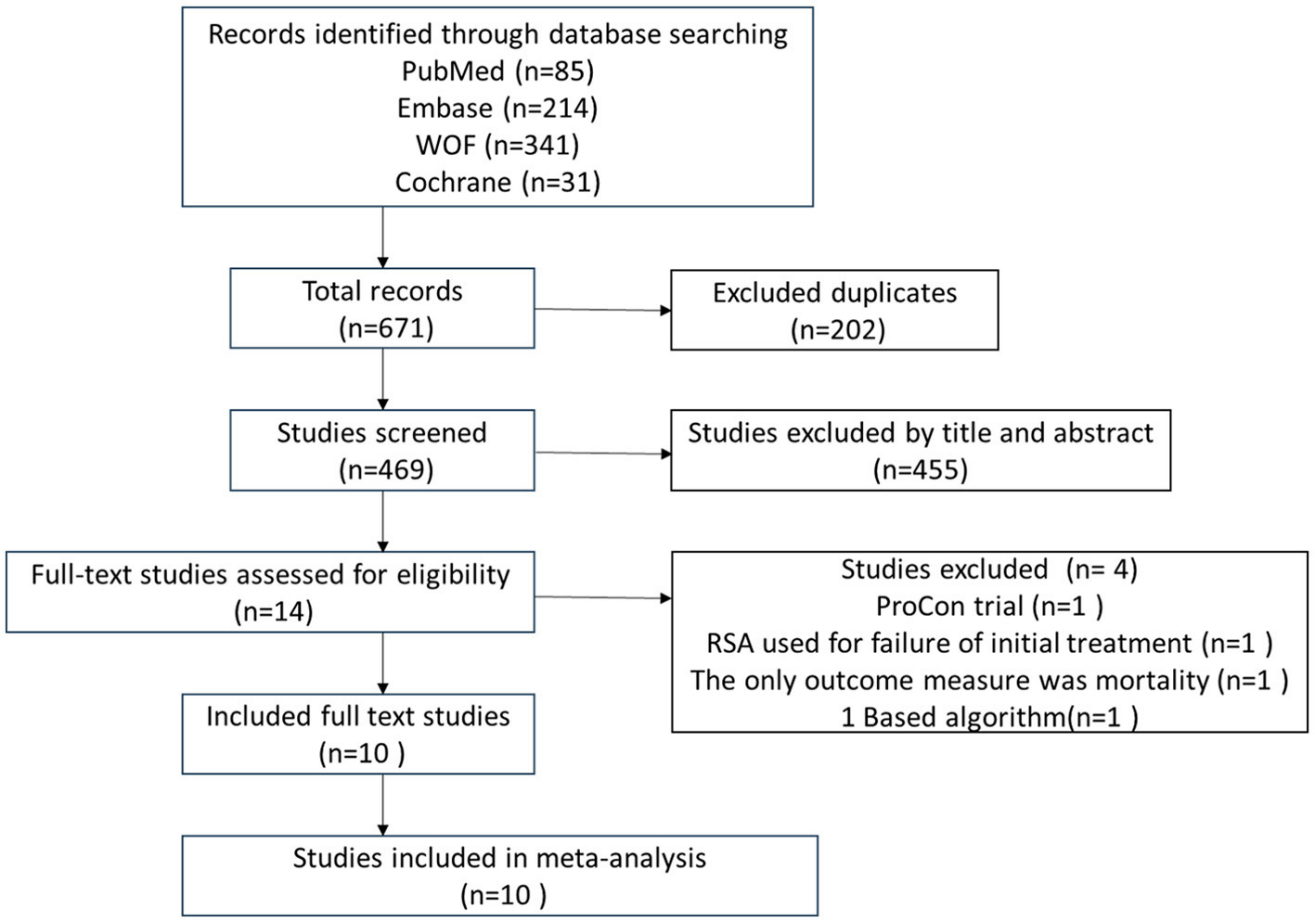
PRISMA flow diagram representing the search and screening process of studies comparing arthroplasty vs. non-surgical treatment of proximal humeral fractures. WOF, Web of Science; Cochrane, Cochrane Library.

A total of 10 studies evaluating arthroplasty vs. non-surgical treatment of PHFs were included in this study (9–18). Figure 1 shows a flowchart of the literature search. The included studies comprised four RCTs and six nRCTs.

### 3.2 Quality assessment

The MINORS scores for all the included studies ranged from 17 to 21, with a median of 18 [interquartile range (IQR), 18–20]. The MINORS score ranged from 17 to 18, with a median of 18 (IQR, 17.75–18) for nRCTs. For RCTs, the scores ranged from 18 to 21, with a median of 20 (IQR, 18.5–20.75). Study-specific MINORS scores are provided in Appendix S2.

### 3.3 Baseline characteristics of study participants

The characteristics of all the 10 studies are summarized in Table 1. There are four RCTs and six nRCTs; three studies compared HA with non-surgical treatment and seven studies compared RSA with non-surgical treatment. These studies provided a total of 508 patients for the meta-analysis: 238 treated operatively and 270 non-operatively. The age of the participants was similar across studies, with a weighted average age of 76.1 years, and 87% of patients were women.

**Table 1:** Baseline characteristics of studies include in the meta-analysis.

**Study design**	**References**	**Study period**	**Neer**	**Description of treatment**	**Female (%)**	**Mean age (years)**	**Sample size**	**Follow-up (months)**
RCT	(9)	2004–2009	4	HA	24 (96)	76.4 (5.6)^‡^	25	12
				Immobilizer for 6 weeks	23 (92)	79.9 (7.7) ^‡^	25	
	(10)	2014–2018	34	RSA	25 (86)	82 ± 3.4^‡^	29	12
				Sling for 3 weeks	26 (87)	85 ± 4.8^‡^	30	
	(11)	2003–2008	4	HA	23 (85)	75.8 (58–90)^†^	27	24
				Sling for 2 weeks	24 (86)	77.5 (60–92)^†^	28	
	(12)	1970–1981	4	HA	12 (75)	65.6 (52–88)	16	>18
				Non-operative	13 (81)	70.1 (60–85)	16	
nRCT	(13)	2016–2019	234	RSA	33 (91.7%)	69.9 (55–89)^†^	28	>12
				Sling for 3–4 week	14 (70%)	72.9 (59–88)^†^	20	
	(14)	2011–2015	34	RSA	22 (78.6)	77 (70–92)^†^	28	>24
				Immobilized for 6 weeks	30 (93.8)	79.2 (70–92)^†^	32	
	(15)	2015–2018	234	RSA	26 (100)	76.8 ± 7.3^‡^	26	12
				Non-operative	41 (91.1)	76.4 ± 7.3^‡^	45	
	(16)	2007–2014	34	RSA	19 (95)	71 (52–88)^†^	20	>24
				Sling for 2 weeks	15 (79)	71 (52–88)^†^	19	
	(17)	2015–2018	4	RSA	23 (95.8%)	77.3 ± 9.5^‡^	24	12
				Non-operative	37 (90.2%)	77.4 ± 10.1^‡^	41	
	(18)	2009–2019	34	HA	9 (60%)	68.5 ± 11.3	15	>12
				Non-operative	3 (21.4%)	77.1 ± 6.5	14	

^†^Mean (range); ^‡^Mean ± SD.

All studies used the Neer classification, including Neer 2-, 3-, and 4-part PHFs for analysis. Most studies (*n* = 5, 50%) used the CMS as the functional outcome. Other measures included VAS (six studies, 60%), forward flexion (eight studies, 80%), external rotation (seven studies, 70%), ASES (three studies, 30%), DASH (three studies, 30%), and ED-5Q (two studies, 20%), all of which were analyzed separately. All studies included information on complications, except for one that did not mention them. Detailed complications are listed in Appendix S3.

### 3.4 Primary outcome measures

**Figure 2:**
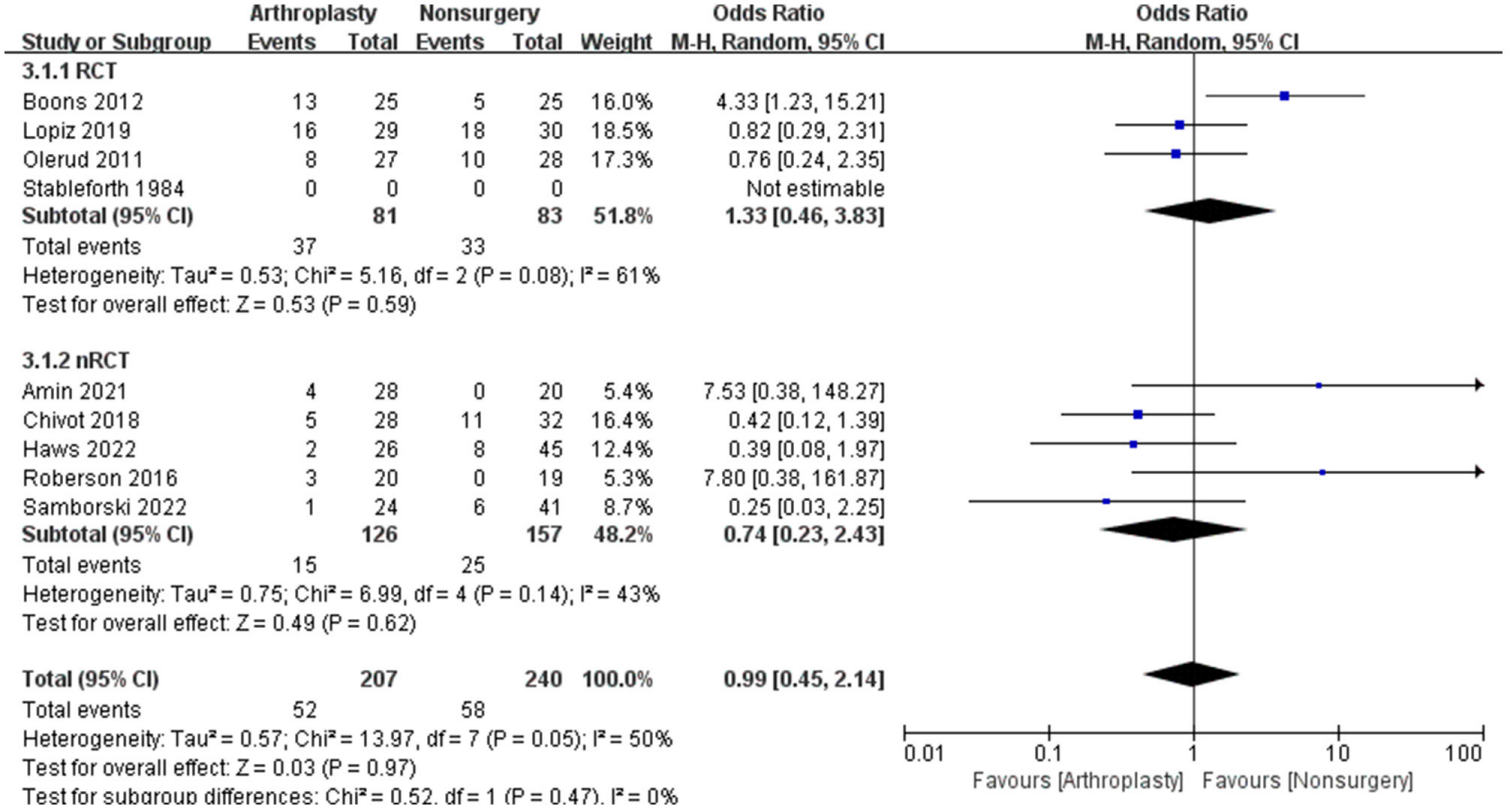
Functional outcome was measured with the Constant-Murley Score in a systematic review of PHFs, comparing arthroplasty with non-operative treatment. SD, standard deviation; IV, inverse variance; CI, confidence interval; RCT, randomized controlled trial.

The CMS is widely used in clinical practice and serves as the primary functional outcome. There are three RCTs and two nRCTs provided CMS data and were included in the evaluation. Figure 2 shows the forest plot for the difference in mean values between surgical and non-surgical treatments. The CMS score showed no difference between arthroplasty and non-surgical treatment, with a mean difference of 2.82 (95% CI = −0.49 to 6.14, *P* = 0.10, *I*^2^ = 77%).

### 3.5 Secondary outcome measures

**Figure 3:**
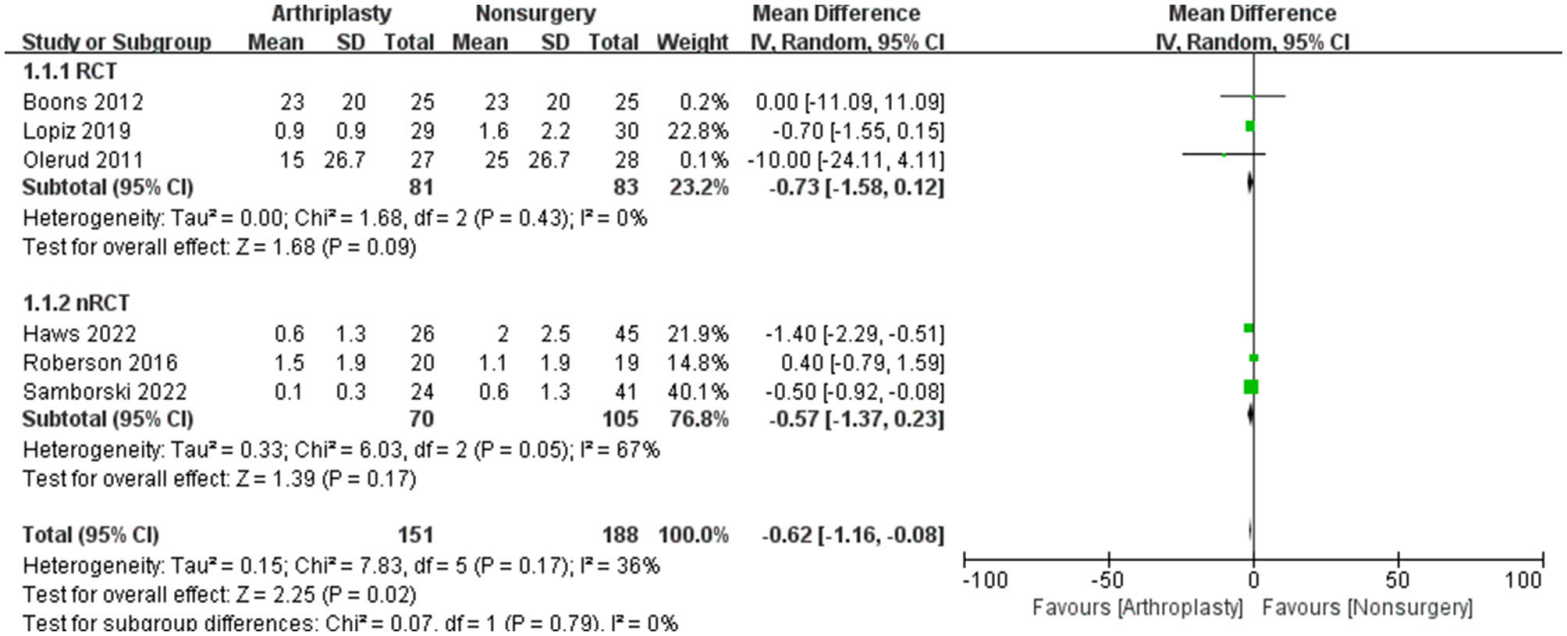
The outcome was measured with VAS in a systematic review of PHFs comparing arthroplasty vs. non-operative treatment. SD, standard deviation; IV, inverse variance; CI, confidence interval; RCT, randomized controlled trial.

There are six studies contained VAS data, and their forest plot for the mean difference was −0.62 (95% CI = −1.16 to −0.08, *P* = 0.02, *I*^2^ = 36%), which is shown in Figure 3. Other data, including forward flexion, external rotation, DASH, ASES, and ED-5Q, were analyzed separately and are shown in Table 2.

**Table 2:** Functional outcome included in the meta-analysis of PHFs comparing arthoplasty vs. non-surgical treatment.

**Outcomes**	**Date**	**Studies**	**Mean difference**	**95% CI**	***p***	***I* ^2^**
Range of motions	Forward flexion	8	11.45	−3.95, 26.85	0.15	89
	External rotation	7	4.22	−2.23, 10.66	0.20	82
Function outcomes	DASH	3	−2.11	−12.76, 8.54	0.70	80
	ASES	3	0.40	−40.40, 41.19	0.98	88
Quality of life	ED-5Q	2	0.09	−0.04, 0.21	0.17	74

DASH, disabilities of the arm, shoulder and hand; ASES, American Shoulder and Elbow Surgeons shoulder; ED-5Q, EuroQol 5 Dimensions Questionnaire.

There are nine studies containing complication information for arthroplasty and non-surgical treatment of PHFs were analyzed, showing no significant difference (mean difference 1.08; 95% CI = 0.51 to 2.25, *P* = 0.85, *I*^2^ = 47%).

A summary of complications among the included studies is shown in Appendix S3. In the arthroplasty group, 9 (4.0%) patients required another surgery, and 36 patients (16.1%) experienced non-anatomic healing or resorption of the greater tuberosity. In the non-surgery group, 10 patients (3.9%) had non-union, and 29 patients (11.3%) developed osteonecrosis, but none of them required additional surgery.

There were four cases (1.8%) of nerve injury in the arthroplasty group, including two cases (0.9%) of suprascapular nerve, one case (0.45%) of radial nerve, and one case (0.45%) of hand paresthesia, all occurring in the RSA subgroup. The articles did not provide further details on the results of these nerve injuries. The surgical infection rate was 0.9%, consisting of one case of continuing sepsis and one case of hematogenous infection.

**Figure 4:**
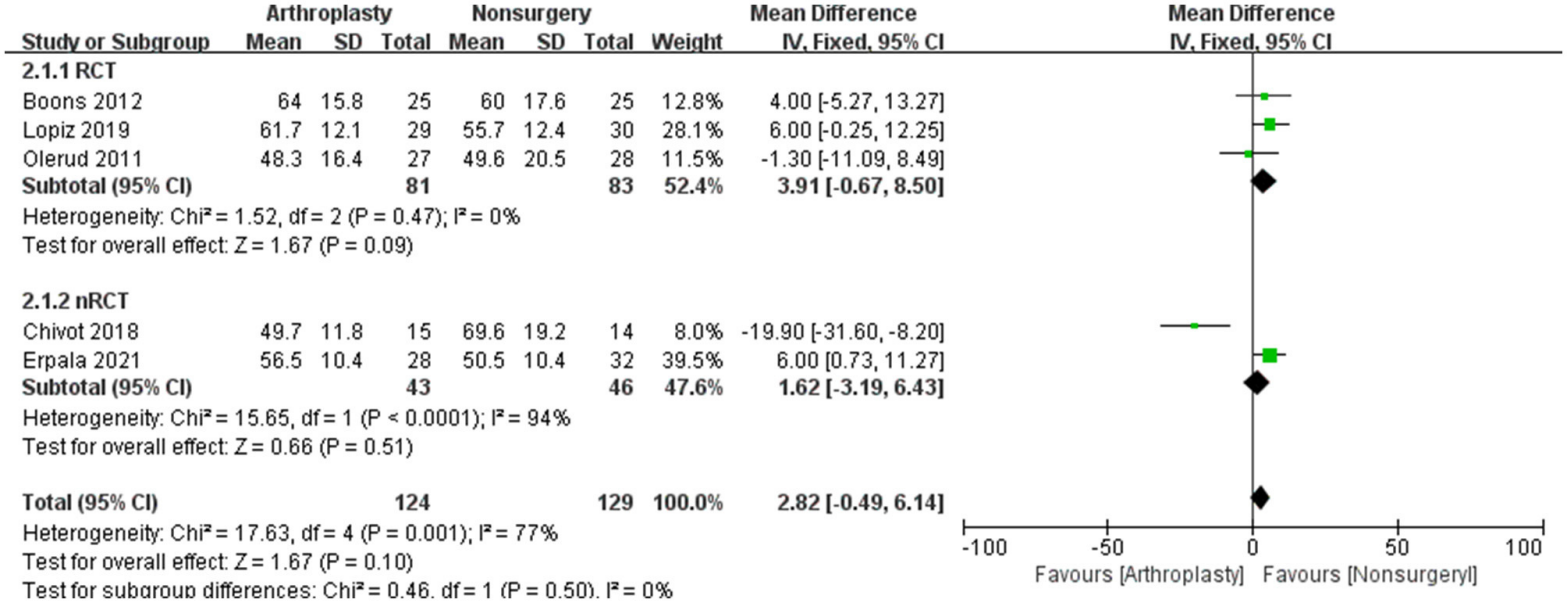
The outcome of complications in a systematic review of PHFs comparing arthroplasty vs. non-operative treatment. SD, standard deviation; IV, inverse variance; CI, confidence interval; RCT, randomized controlled trial.

The incidence of complications was higher in the surgery group (25.1%) compared to the non-surgical group (24.2%), with an OR of 0.99 (95% CI, 0.45–2.14; *I*^2^ = 50%), but this difference was no significant (*Z* = 0.03, *P* = 0.97) (Figure 4). However, the complication rate for HA (35.3%) was significantly higher than that for RSA (20%).

### 3.6 Sensitivity analysis and subgroup analysis

The results of the sensitivity and subgroup analyses for RCT and nRCT were mostly consistent with the original results (Table 3). To further explore the efficacy of arthroplasty, we compared RSA and HA separately with non-surgical treatment and found that RSA resulted in better CMS and VAS score, while HA showed no significant difference. Studies with high consistency using a fixed-effects model indicated that arthroplasty could result in better functional outcomes, pain relief, and range of motion compared to non-surgical management.

**Table 3:** Subgroup and sensitivity analyses of studies included in the meta-analysis of PHFs comparing arthoplasty vs. non-surgical treatment.

**Analysis description**	**Constant score**			**VAS**			**Forward flexion**			**External**			**Complications**		
	***n***	**MD (95% CI)**	***P*** **-value**	***n***	**MD (95% CI)**	***P*** **-value**	***n***	**MD (95% CI)**	***P*** **-value**	***n***	**MD (95% CI)**	***P*** **-value**	***n***	**MD (95% CI)**	***P*** **-value**
All studies	5	2.82 (−0.49, 6.14)	0.10	6	−0.62 (−1.16, −0.08)	0.02	8	11.45 (−3.95, 26.85)	0.15	7	4.22 (−2.23, 10.66)	0.20	9	1.08 (0.51, 2.25)	0.85
RCT	3	3.91 (−0.67, 8.50)	0.09	3	−0.73 (−1.58, 0.12)	0.09	2	−0.93 (−19.63, 17.76)	0.92	1	−2.00 (−4.33, 0.33)	0.09	4	1.48 (0.59, 3.72)	0.40
nRCT	2	1.62 (−3.19, 6.43)	0.51	3	−0.57 (−1.37, 0.23)	0.17	6	14.01 (−3.78, 31.80)	0.12	6	6.53 (0.85, 12.22)	0.02^*^	5	0.74 (0.23, 2.43)	0.62
RSA	2	6.0 (1.97, 10.03)	0.00^*^	4	−0.61 (−1.17, −0.05)	0.03^*^	3	−14.95 (−43.66, 13.77)	0.31	5	8.52 (5.08, 11.95)	0.00^*^	6	0.71 (0.31,1.63)	0.42
HA	3	−5.32 (18.66, 8.02)	0.43	2	−4.01 (−13.61, 5.60)	0.41	5	23.11 (8.17, 38.04)	0.00^*^	2	−12.27 (−38.71, 14.18)	0.36	3	2.00 (0.56, 7.13)	0.28
Fixed-effects model	4	4.81 (1.35, 8.27)	0.01^*^	6	−0.59 (−0.92, −0.26)	0.00^*^	4	5.73 (−2.98, 14.43)	0.00^*^	5	8.53 (5.08, 11.95)	0.00^*^	9	1.04 (0.66, 1.64)	0.86

MD, mean difference; CI, confidence interval.

^*^Significant pooled effect (*P* < 0.05).

## 4 Discussion

Based on the results of this meta-analysis, arthroplasty treatment can achieve better pain relief than non-surgical treatment, but the functional outcomes and complication rates are not significantly different from non-surgical treatment. However, the results of the subgroup analysis showed that RSA could achieve better functional scores and ROM than non-surgical treatment. Interestingly, most of the studies with high consistency indicated that arthroplasty was associated with better clinical outcomes than non-surgical treatment.

RSA, HA, and conservative treatments are widely used in the treatment of PHFs. However, there is still no consensus on the optimal treatment approach. To date, the clinical outcomes of arthroplasty vs. non-surgical treatment have been widely studied. Some studies favor arthroplasty treatment (19–21) or conservative treatment (22–25), while others find no difference (4, 5, 26–28).

Chen et al. (19) concluded that the ranking of treatments in terms of high CMS was RSA, ORIF, intramedullary nailing (IN), non-operative treatment (NOT), and HA. The ranking for reducing the total incidence of complications was RSA, NOT, HA, IN, and ORIF. Du et al. (20) reported that the constant scores were ranked as follows: RSA, HA, NOA, and ORIF. The overall reoperation reduction levels were ranked as RSA, NOA, HA, and ORIF.

Iyengar et al. (22) conducted a systematic review of 12 studies involving 650 patients and found that the conservative treatment of 1- or 2-part fractures resulted in a 100% radiographic union rate and good mobility recovery. Radiographic bone union rates of 98% were also achieved for 3- or 4-part fractures, with a complication rate of only 13%.

Wu et al. (21) analyzed a private payment claims database of 22 million patient records and found that the surgical treatment of PHFs was associated with significantly higher rates of complications, reoperation, and length of hospital stay, resulting in significantly higher treatment costs.

To the best of our knowledge, there have been few studies examining arthroplasty vs. non-surgical treatment for PHFs. A systematic analysis comparing arthroplasty and conservative treatment for PHFs, which included 33 articles involving 1,096 patients, found that arthroplasty treatment resulted in higher CMS scores than non-surgical treatment (29). However, the authors concluded that this result could be attributed to selection bias, fracture classification differences, and variations in scoring criteria, as multiple regression analyses showed the opposite result.

RSA is increasingly used in treating PHFs, and its clinical effectiveness has been widely validated (30, 31). The introduction of the RSA provides a better option for the treatment of complex PHFs in older adults. Although the long-term durability of this prosthesis is still unknown, the midterm results are satisfactory (32). Several studies have shown that RSA has better clinical outcomes, fewer complications, and lower reoperation rates than other surgical treatments (33–36).

Nwachukwu et al. (37) found that, compared with non-operative management, both HA and RSA can be cost-effective strategies for managing complex PHFs. This study also found that RSA could achieve better CMS scores than non-surgical treatment. Although only two studies were included, they were of high quality and high consistency.

Complications of RSA, which range from 17 to 75%, include instability, scapular notch, nerve injury, infection, hematoma, acromion/scapular stress fracture, intraoperative fracture of the humerus and glenoid, loosening of the glenoid basal plate or humeral stem, deltoid fatigue, and complex regional pain syndrome (30, 32, 38). In an analysis of 132,005 hospitalized patients aged 65 years and older with a proximal humeral fracture, the overall incidence of adverse events during hospitalization was 21%, with the risk of adverse events for arthroplasty being 4.4 times higher than that for non-surgical treatment (39).

HA is especially controversial in the treatment of PHFs, as studies have shown that HA does not achieve better results (40). Its functional outcome is directly related to the healing of the tuberosity and rotator cuff (40). In contrast, RSA can be used in the absence of a rotator cuff, and its functional outcome is not dependent on the anatomical reduction and healing of the greater tubercle (38). Although HA can preserve more joint components to some extent, its functional outcome is controversy. And RSA revision surgery can be performed in case of HA failure.

Patients who undergo initial periods of non-operative management have worse functional outcomes and higher complication rates than those who undergo acute RSA for PHFs (41). Compared with primary shoulder arthroplasty and revision shoulder arthroplasty, primary RSA can achieve better functional results (38, 42). A recent study confirmed that acute RSA results in better clinical outcomes, a better range of motion, and a lower complication rate than RSA performed secondary to conservative or surgical management (43).

An important factor in the successful management of a proximal humeral fracture is not only adequate surgical capacity but also the decision to undergo surgical or conservative treatment (44). Treatment decisions should not be based solely on the Neer classification, as it may have less clinical importance than previously assumed (45). Fracture type and radiographic appearances do not always correspond with functional results (46). The physiological state of the patient, the severity of the fracture pattern, and the experience and competence of the surgeon are three major factors that should be considered when choosing the appropriate treatment (1). Spross et al. (47) developed a comprehensive algorithm as a non-compulsory treatment guideline for PHF, which has proven helpful for decision-making and achieving satisfying results.

Our systematic review of the literature found that the major complications of conservative treatment for PHFs were avascular osteonecrosis and non-union. Soler-Peiro et al. (23) found that the most frequent complication of conservative treatment was malunion (21%), followed by avascular necrosis (9%). A meta-analysis comparing surgical treatment and non-surgical treatments for displaced PHFs found no significant difference in clinical outcomes between the two approaches, which is consistent with our results.

However, they noted that the overall complication rate was 3.3 times higher following surgical treatment (27). Factors leading to non-union of PHFs mainly include displaced 2-part fractures, smoking, persistent glenohumeral arthritis or rheumatoid arthritis, OTA B2.3 and C2.3 fractures, and comminution (48).

To the best of our knowledge, this study is the first to compare arthroplasty with non-surgical treatment for PHFs. The direct results of this meta-analysis showed no significant difference in clinical outcomes and complications between arthroplasty and non-surgical treatment. However, sensitivity analysis indicated that the clinical outcomes of arthroplasty were better than those of non-surgical treatment. In addition, RSA was found to achieve better functional scores.

The main complications of arthroplasty include malunion or non-union of the greater tuberosity, wound infection, and nerve injury. In contrast, the complications of non-operative treatment mainly include fracture non-union, ischemic necrosis of the humeral head, and traumatic arthritis.

The limitations of this study is the small number of RCTs (only four) and nRCTs (only six) included in the analyses. More case reports should be identified and included in the analyses. As with all meta-analyses, there are inherent limitations, such as the heterogeneity of the included studies, missed studies in our search, and unknown biases in the original literature. A random-effects model was selected to control for some of the inherent heterogeneity, which could, to some extent, affect the credibility of the results. Additionally, including only published studies may introduce publication bias, and limiting the search to the English language may have excluded some potentially relevant studies.

## 5 Conclusion

Based on the results of the meta-analysis of existing studies, it is believed that there were no significant differences in complications between arthroplasty and non-surgical treatment for PHFs. RSA could achieve better functional results than non-surgical treatment, while HA could only achieve better forward flexion.

## Data availability statement

The original contributions presented in the study are included in the article/Supplementary material, further inquiries can be directed to the corresponding author.

## Author contributions

BL: Conceptualization, Data curation, Investigation, Methodology, Software, Supervision, Writing – original draft, Writing – review & editing. SZ: Data curation, Software, Writing – original draft. JP: Methodology, Writing – review & editing. AL: Supervision, Writing – review & editing. DG: Visualization, Writing – review & editing. ZP: Formal analysis, Writing – original draft. QF: Conceptualization, Investigation, Writing – original draft, Writing – review & editing.
