# Supplementary material for: Comparison between arthroplasty and non-operative treatment for proximal humeral fractures: a systematic review and meta-analysis
Source: Front Med (Lausanne). 2024 Sep 6;11:1436000. doi: 10.3389/fmed.2024.1436000 (PMC11413808; doi:10.3389/fmed.2024.1436000)

Appendix 1 The search strategy of 3 electronic databases (Pubmed, Embase, Web of science)

**Pubmed**

#1 (((Shoulder Fractures [Mesh]) OR (((proximal humeral fractures [Title/Abstract])) OR (shoulder fracture [Title/Abstract])))

#2 ((Conservative Treatment [Mesh]) OR (((conservative treatment [Title/Abstract])) OR (nonoperative treatment [Title/Abstract]))))

#3 ((((((hemiarthroplasty [Title/Abstract])) OR (reverse shoulder arthroplasty [Title/Abstract])) OR (arthroplasty [Title/Abstract])) OR (Hemiarthroplasty [Mesh])) OR (Arthroplasty, Replacement [Mesh]))

#1 AND #2 AND #3


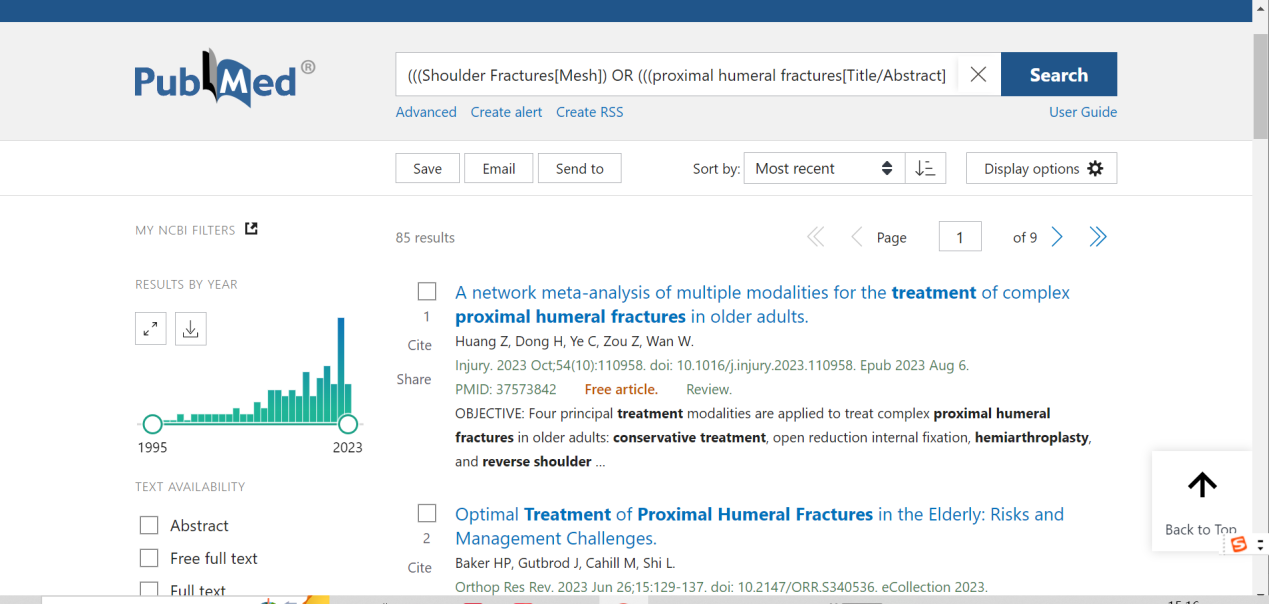


**Embase**

Session Results

No. Query Results Results Date

#10. #3 AND #6 AND #9 214 5 May 2023

#9. #7 OR #8 142,791 5 May 2023

#8. hemiarthroplasty:ab,ti OR 'reverse shoulder 97,300 5 May 2023

arthroplasty':ab,ti OR arthroplasty:ab,ti

#7. 'hemiarthroplasty'/exp OR 'arthroplasty'/exp 121,972 5 May 2023

#6. #4 OR #5 755,825 5 May 2023

#5. 'conservative treatment':ab,ti OR 'nonoperative 52,696 5 May 2023

treatment':ab,ti

#4. 'conservative treatment'/exp 730,175 5 May 2023

#3. #1 OR #2 5,916 5 May 2023

#2. 'proximal humeral fractures':ab,ti OR 'shoulder 1,854 5 May 2023

fracture':ab,ti

#1. 'shoulder fracture'/exp 5,032 5 May 2023


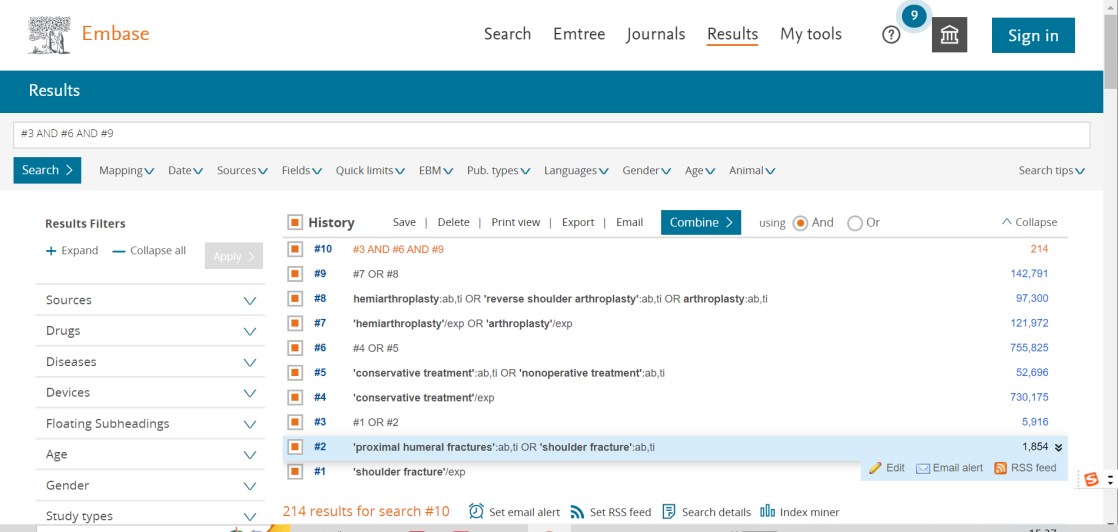


**Cochrane**

Date Run: 05/05/2023 15:54:07

Comment:

ID Search Hits

#1 MeSH descriptor: [Shoulder Fractures] explode all trees 181

#2 (proximal humeral fractures):ti,ab,kw OR (shoulder fracture):ti,ab,kw (Word variations have been searched) 1453

#3 #1 or #2 1467

#4 MeSH descriptor: [Conservative Treatment] explode all trees 650

#5 (conservative treatment):ti,ab,kw OR (nonoperative treatment):ti,ab,kw (Word variations have been searched) 15600

#6 MeSH descriptor: [Hemiarthroplasty] explode all trees 96

#7 MeSH descriptor: [Arthroplasty] explode all trees 7034

#8 (hemiarthroplasty):ti,ab,kw OR (reverse shoulder arthroplasty):ti,ab,kw OR (arthroplasty):ti,ab,kw (Word variations have been searched) 15353

#9 #4 OR #5 15600

#10 #6 OR #7 OR #8 16124

#11 #3 AND #9 AND #10 31


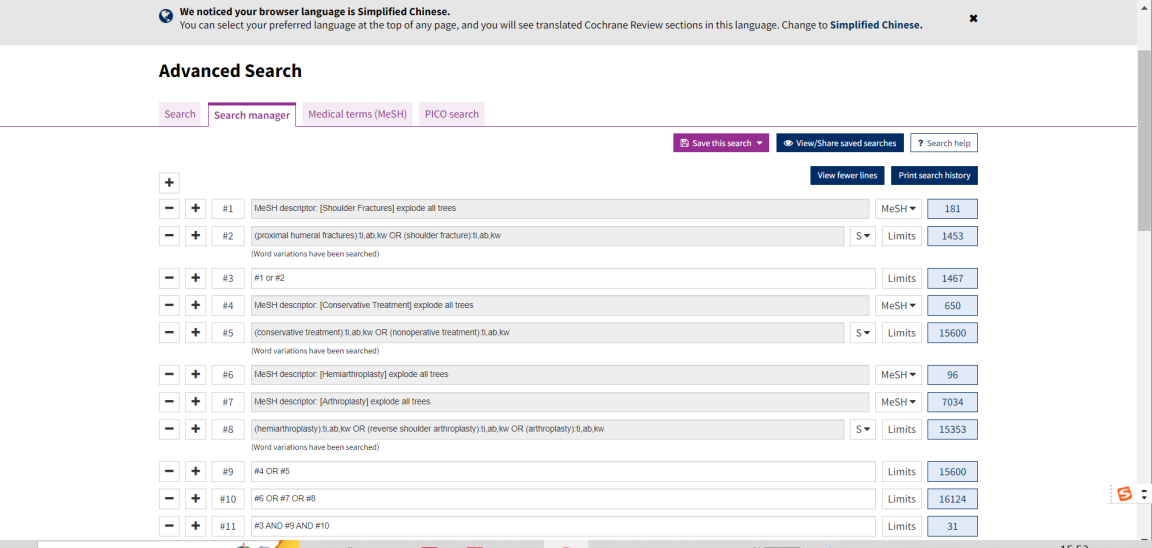


**Web of Science**

# Database: All Databases

# Entitlements:

- WOS: 1985 to 2023

- KJD: 1980 to 2023

- MEDLINE: 1950 to 2023

- PPRN: 1991 to 2023

- PQDT: 1637 to 2023

- SCIELO: 2002 to 2023

# Searches:

1: (TS=(proximal humeral fractures)) OR TS=(shoulder fracture) and Preprint Citation Index (Exclude – Database) Date Run: Sun May 5 2023 11:52:06 GMT+0800 Results: 17528

2: (TS=(conservative treatment)) OR TS=(nonoperative treatment) and Preprint Citation Index (Exclude – Database) Date Run: Sun May 5 2023 11:52:39 GMT+0800 Results: 115466

3: ((TS=(hemiarthroplasty)) OR TS=(reverse shoulder arthroplasty)) OR TS=(arthroplasty) and Preprint Citation Index (Exclude – Database) Date Run: Sun May 5 2023 11:53:19 GMT+0800 Results: 139076

4: #3 AND #2 AND #1 and Preprint Citation Index (Exclude – Database) Date Run: Sun May 5 2023 11:53:27 GMT+0800 Results: 341


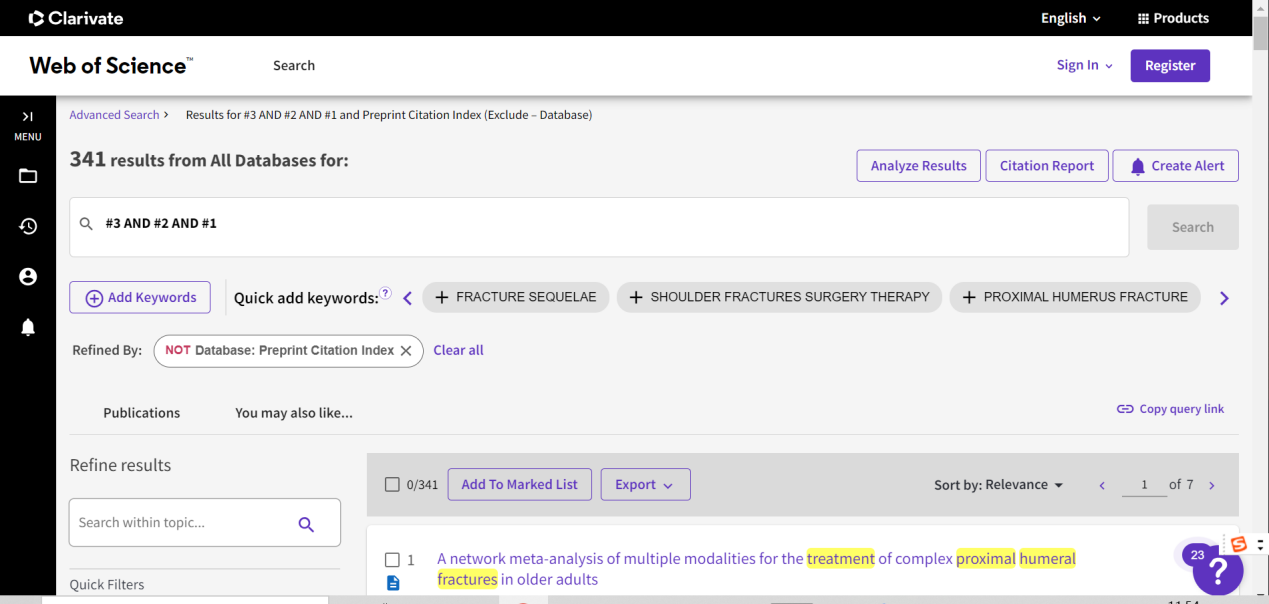

Supplement: Supplementary file 1 [file Table_1.DOCX]
