# Supplementary material for: Comparison between arthroplasty and non-operative treatment for proximal humeral fractures: a systematic review and meta-analysis
Source: Front Med (Lausanne). 2024 Sep 6;11:1436000. doi: 10.3389/fmed.2024.1436000 (PMC11413808; doi:10.3389/fmed.2024.1436000)
Supplement: Supplementary file 2 [file Table_2.DOCX]

Appendix S2: Methodological Index for Non-Randomized Studies (MINORS)

| Items | Amin 2021 | Chivot 2018 | Haws 2022 | Roberson 2016 | Samborski 2022 | Erpala 2021 | Boons 2012 | Lopiz 2019 | Olerud 2011 | Stableforth 1984 |
| --- | --- | --- | --- | --- | --- | --- | --- | --- | --- | --- |
| 1. A stated aim of the study | 2 | 2 | 2 | 2 | 2 | 2 | 2 | 2 | 2 | 0 |
| 2. Inclusion of consecutive patients | 2 | 2 | 2 | 2 | 2 | 2 | 2 | 2 | 2 | 2 |
| 3. Prospective collection of data | 2 | 2 | 2 | 2 | 2 | 2 | 2 | 2 | 2 | 2 |
| 4. Endpoint appropriate to the study aim | 2 | 2 | 2 | 2 | 2 | 2 | 2 | 2 | 2 | 2 |
| 5. Unbiased evaluation of endpoints | 0 | 0 | 0 | 0 | 0 | 0 | 0 | 0 | 0 | 0 |
| 6. Follow-up period appropriate to the major endpoint | 2 | 2 | 2 | 2 | 2 | 2 | 2 | 2 | 2 | 2 |
| 7. Loss to follow up not exceeding 5% | 0 | 0 | 0 | 0 | 0 | 0 | 0 | 2 | 0 | 2 |
| 8. A control group having the gold standard intervention | 0 | 0 | 0 | 0 | 0 | 0 | 2 | 2 | 2 | 0 |
| 9. Contemporary groups | 2 | 2 | 2 | 2 | 2 | 2 | 2 | 2 | 2 | 2 |
| 10. Baseline equivalence of groups | 2 | 2 | 2 | 2 | 2 | 2 | 2 | 2 | 2 | 2 |
| 11. Prospective calculation of the sample size | 2 | 2 | 2 | 2 | 1 | 2 | 2 | 1 | 2 | 2 |
| 12. Statistical analyses adapted to the study design | 2 | 2 | 2 | 2 | 2 | 2 | 2 | 2 | 2 | 2 |
| total points | 18 | 18 | 18 | 18 | 17 | 18 | 20 | 21 | 20 | 18 |
