# Supplementary material for: Comparison between arthroplasty and non-operative treatment for proximal humeral fractures: a systematic review and meta-analysis
Source: Front Med (Lausanne). 2024 Sep 6;11:1436000. doi: 10.3389/fmed.2024.1436000 (PMC11413808; doi:10.3389/fmed.2024.1436000)
Supplement: Supplementary file 3 [file Table_3.DOCX]

Appendix S3 Complications include in the meta-analysis

| study design | 1st author (year) | Group | Additional surgery, reason | Nonunion | Osteonecrosis | Others |
| --- | --- | --- | --- | --- | --- | --- |
| RCT | Boons 2012 | Surgery | 1(4%) revision surgery, head-stem separation | 2(8%) | 0 | 4(16%), malposition of the greater tuberosity  5(20%), Secondary superior migration of the greater tuberosity  1(4%), proximal migration of the hemiarthroplasty |
|  |  | Non-surgery | 0 | 3(12%) | 2(8%) | 0 |
|  | Lopiz 2019 | Surgery | 0 | 0 | 0 | 2(6.9%), suprascapular nerve injury  14 (48%), GT nonanatomic healing or resorption |
|  |  | Non-surgery | 0 | 1 (3.4%) | 17 (58.6%) | 0 |
|  | Olerud 2011 | Surgery | 1(3.7%) implant removed, screw penetration  1(3.7%) acromioplasty and release of adhesions, impingement and stiffness  1(3.7%) greater tubercle reduction and refixation, displacement of the greater tubercle | 0 | 0 | 5(19%), greater tubercle dislocation or resorption |
|  |  | Non-surgery | 1(4%), complete displacement of the shaft | 1(4%) | 3(11%) | 5(19%), posttraumatic osteoarthritis |
|  | Stableforth 1984 | Surgery | 1(6.3%) prosthesis removed, continuing sepsis | 0 | 0 | 1(6.3%), hematomata  1(6.3%), death for other reason |
|  |  | Non-surgery | 0 | 0 | 0 | 1(6.3%), death for other reason |
| nRCT | Amin 2021 | Surgery | 0 | 0 | 0 | 1(3.6), hand paresthesia  3(10.7), severe heterotopic bone formation |
|  |  | Non-surgery | 0 | 0 | 0 | 0 |
|  | Chivot 2018 | Surgery | 1(3.6), hematogenous infection | 0 | 0 | 1(3.6%), traumatic dislocation  3(10%), greater tuberosity resorption |
|  |  | Non-surgery | 0 | 1(3%) | 3(9%) | 4(12%), secondary displacement  3(9%), resorption |
|  | Haws 2022 | Surgery | 0 | 0 | 0 | 1(3.8%), periprosthetic humerus fractures  1(3.8%), musculocutaneous/radial nerve palsy |
|  |  | Non-surgery | 4(8.9%) | 3(6.7%) | 1(2.2%) | 0 |
|  | Roberson 2016 | Surgery | 3(15%) | 0 | 0 | 0 |
|  |  | Non-surgery | 0 | 0 | 0 | 0 |
|  | Samborski 2022 | Surgery | 0 | 0 | 0 | 1(4.2%), periprosthetic fracture |
|  |  | Non-surgery | 2(4.9%) | 1(2.4%) | 3(7.3%) | 0 |
